# Debate: Young people are living in unprecedented times – too much chaos or too little resilience?: an argument to talk less about resilience

**DOI:** 10.1111/camh.70085

**Published:** 2026-03-12

**Authors:** Michael Ungar

**Affiliations:** ^1^ Canada Research Chair in Child, Family and Community Resilience Halifax NS Canada; ^2^ Dalhousie University Halifax NS Canada

**Keywords:** Resilience, developmental psychopathology, social environment, psychopathology, adaptive behavior

## Abstract

Has the concept of childhood resilience become too common, its meaning obscured by its overuse? This paper provides an argument for more constrained use of the term resilience, identifying the resulting problem of young people pathologizing normative risk exposure (a concept referred to as disorderism). Instead, resilience science emphasizes the need for exposure to manageable amounts of risk for optimal development. Indeed, studies of resilience have shown that most children demonstrate resilience because of the large networks of supports they are able to access when stressed.

Today's children are being exposed to a toxic soup of psychological and social stressors, ranging from excessive use of screens and social media to ecological nihilism, identity politics, and economic uncertainty affecting their families and communities. Rates of mental disorder suggest long and enduring consequences (Canadian Institute for Healthcare Information, 2025).

Are all potentially traumatizing experiences (PTE) (Bonanno & Mancini, 2012) likely to contribute to psychological disorder? Or is our capacity for resilience greater than our susceptibility to becoming fragile? Bonnano and Mancini use the concept of PTE to distinguish between an exposure to stress and its possible, but uncertain, consequences. This avoids the falsehood that every stressful experience is likely to create trauma‐induced symptoms. In fact, these symptoms are relatively uncommon. Decades of resilience research show that most children are likely to have the internal and external capacities to withstand age‐appropriate stressors when those stressors are defined as normal and expected in a particular context or culture (Masten, 2025). We assume, though, that children must be shielded from the negative consequences of all stress and adversity. The result is children being labelled as vulnerable when they need not be.

An excessive focus on trauma overlooks the fact that with even minimal amounts of support, children are able to sustain themselves and grow their way out of a crisis. Even studies of children who have experienced unusual challenges, like gender‐diverse young people experiencing homelessness (Shelton, Wagaman, Small, & Abramovich, 2018), show that with just a small network of social supports, some emotional regulation strategies, and when possible, access to healthcare and social services, successful development is achievable. Even online communities may buffer the impact of stress when children participate in relationships that value their contributions (Boers, Afzali, Newton, & Conrod, 2019). In other words, resilience is the default growth pattern following stress; fragility is the exception.

There is, however, one critically important caveat to this optimism. When children are part of communities that are structurally and socially marginalized because of race, ethnicity, ability, sex, gender or migration status, or they are facing the very complicated and entwined risk factors that follow political violence, climate change, and forcible displacement, then traumatic events are far more likely to predict mental disorders and problem behaviors. The issue, however, is not the lack of human capacity to heal but the very real and oppressive conditions surrounding some (but not all) children, which makes them vulnerable.

Just as importantly, an excessive focus on trauma overlooks a child's need for exposure to age‐appropriate normative stressors that can result in periods of social exclusion, failure, and even non‐life‐threatening injury that occurs in the context of risky play (Brussoni et al., 2015). An ever‐increasing tendency to be overprotective and label children's discomfort as problems extends to even normative developmental stressors like changing schools as one gets older, the death of a cherished pet or grandparent, failure to succeed in a sport, and minor bullying. None of these should be insurmountable barriers to growth for most children who have access to resilience‐enabling resources like a positive sense of self‐worth, a small group of positive relationships and institutional support at school and in their community (Morris & Hays‐Grudo, 2023). Indeed, these events, though disheartening, are opportunities for development that produce reasonable and temporary periods of psychological distress that should not be pathologized.

Instead, our children's resilience is being undermined by a perception of their vulnerability and need for formal treatment, a pattern sometimes referred to as ‘disorderism’ (van Hulst, Groen‐Blokhuis, de Ridder, & Dekkers, 2025). This fragility is then internalized and can become part of a child's enduring identity. Within a trauma‐discourse, children are encouraged to find ways to avoid (or worse, clinically treat) the very common experience of feeling depressed for a short time, hating their bodies for a short time, being lonely and feeling excluded for a short time, and so on. To even suggest that these experiences are developmentally appropriate building blocks for future resilience has become antithetical to the practice of mental health. Unfortunately, providing a trauma specialist for a child experiencing a normative stressor may, even with good intentions, prove to be iatrogenic.

Unfortunately, our efforts to destigmatize mental health disorders and to educate children to seek help when necessary may have had an unintended consequence. They are self‐diagnosing without the clinical experience to distinguish the common from the exceptional. When this occurs on a large scale, and when children misapply labels to themselves (or have those labels attached to them by their caregivers and educators), those individuals with very real experiences of PTE are less likely to access the resources necessary to help as helping systems are flooded with the false positives of overdiagnosis.

Furthermore, by giving all children the same access to treatment, there is an implied equivalency between a child who has been exposed to extreme adversities like intergenerational trauma related to structural violence and a child who is given an accommodation because of a normative experience of loss. This emphasis on accommodations is just one dimension of the narrative of fragility that is undermining resilience. Here again, one needs to be cautious and make the distinction between an accommodation for a child's disability or disorder that is clinically valid and justification for an evidence‐informed approach to promoting successful development versus a child who is stressed but likely to recover with the support of those already in their lives.

As we grow in our awareness of adversity around the world, we are going to need a better, more dynamic understanding of vulnerability and resilience. They are not opposite ends of a single continuum. As Keyes (2024) explains, the opposite of resilience and thriving is languishing, not vulnerability. Vulnerability is a second and separate dimension of wellbeing, with extreme vulnerability resulting from a combination of individual challenges (like dyslexia, exposure to domestic violence, etc.) and external stressors (chronic poverty, psychological disorder, racism, etc.). We mistakenly combine these two dimensions into one. One can be extremely vulnerable and their exposure to adversity continue but still have the internal and external resources to cope with a challenging situation.

Recent research on these two aspects of mental health is demonstrating this need for two‐dimensional thinking. For example, in a study of over 40,000 Chinese adolescents that investigated the many different risk and protective factors associated with positive youth development and psychological symptoms, there was a statistically significant stress alleviating function of positive factors in an adolescent's life (Huang et al., 2026). In fact, positive experiences had a much bigger influence on a youth's experience of mental health than their psychological symptoms. Put simply, self‐esteem, emotional regulation, and an adult who cares even a little is going to compensate for the impact of symptoms related to depression and anxiety, especially as children get older and exposure to adversity increases. Results like this remind us that a fatalistic view that symptoms of mental disorders and their triggers are going to produce bad developmental outcomes and contribute to a child's sense of fragility is not factually true when a child's environment is capable of providing resilience‐enabling supports.

In seeking to understand today's children, it should be assumed that most children have the minimum resources to experience resilience and support positive developmental outcomes. Instead, we seem to be focusing on children's vulnerability and ignoring population differences. Our attention and clinical resources need to be directed toward children who really are experiencing challenges but lack the psychosocial and institutional necessities for optimal development. Without distinguishing children who need interventions from the vast majority that will experience everyday hassles and temporary setbacks in a chaotic and stressful world but in the end do quite well, we will pathologize every child's temporary discomfort while ignoring their potential for resilience.

## Funding information

No funders declared.

## Conflict of interest statement

The author has no conflicts of interest to declare.

## Ethics statement

No ethics approval was required for this debate article.

## Data Availability

Data sharing is not applicable to this article as no data sets were generated or analyzed for this debate article.
